# The incidence and analysis of ipsilateral occult hernia in patients undergoing hernia repair: a single institution retrospective study of 1066 patients

**DOI:** 10.1186/s12893-021-01181-8

**Published:** 2021-04-07

**Authors:** Jun He, Ying-jie Xu, Peng Sun, Jue Wang, Cheng-guang Yang

**Affiliations:** Department of General Surgery, Tongren Hospital, Shanghai Jiao Tong University School of Medicine, 1111 XianXia Road, Shanghai, 200336 China

**Keywords:** Inguinal hernia, Occult hernia, Hernia repair, Lichtenstein, Laparoscopic herniorrhaphy

## Abstract

**Background:**

Misdiagnosis or failure to intraoperatively detect occult hernia in the inguinal region can lead to the recurrence of postoperative hernia and the appearance of local pain symptoms, which affect the patient’s quality of life and make it difficult to reperform hernia repair.

**Methods:**

This study included 1066 inguinal hernia patients who underwent surgical treatment at Shanghai Tongren Hospital between January 2016 and October 2018 to investigate ipsilateral occult hernia epidemiology, to analyze the characteristics of ipsilateral occult hernias with regards to patient age, gender, classification and anatomical site, and to explore the superiority and inferiority of the expert hernia surgeons/ non-expert hernia surgeons group and of operation methods in finding occult inguinal hernias.

**Results:**

The incidence of ipsilateral occult hernia in the surgical population was 8.26%. Ipsilateral occult hernia included indirect inguinal hernia, direct inguinal hernia, femoral hernia, obturator hernia, and spigelian hernia, among which the highest incidence was direct inguinal hernia (4.11%), followed by indirect inguinal hernia (2.45%). There was no difference in the incidence of ipsilateral occult hernia between males and females, but there were significant differences in the incidence of ipsilateral occult hernia, which decreased gradually with increasing age in patients younger than 70 years-old; there was no difference in incidence in patients over 70 years-old. There were significant differences in the incidence of ipsilateral occult hernia in the bilateral inguinal region between direct and femoral hernia, with the higher incidence found on the right side; in contrast, there was no difference in the incidence of indirect inguinal hernia in the bilateral inguinal region. There was no difference in the ability of experienced physicians to detect ipsilateral occult inguinal hernias, either professionally or by surgery.

**Conclusions:**

Ipsilateral occult inguinal hernia has a higher incidence in patients with inguinal hernia, especially older patients; therefore, it is necessary for experienced surgeons to carefully detect for possible occult hernia during the operation and in elderly patients.

## Background

The guidelines for diagnosing and treating symptomatic or apparent inguinal hernia have been clearly stated [1], and the techniques for diagnosis and treatment are no longer a problem. However, with the rise in surgical patients, experienced surgeons will occasionally find two or more kinds of hernia in the ipsilateral or contralateral inguinal regions after preoperative examinations that could only preliminarily ascertain that there was one kind of hernia, these cases often represent occult hernias [2–4]. Once the occult hernia is found intraoperatively, it can be properly handled in time. However, if the surgeon lacks experience or does not understand the morbidity associated with these cases, they are likely to miss the hidden inguinal hernia, leading to postoperative hernia recurrence or local adverse reactions, such as chronic pain that negatively impacts the patient's quality of life. At the same time, due to the occurrence of local anatomical structural disorders or local dense adhesion after surgery, especially when combined with cardiopulmonary dysfunction, the difficulty and risk of reoperation are increased [5, 6]. Therefore, attention should be paid to the existence of ipsilateral or contralateral occult hernia. Currently, most of the literature reports on occult hernia are the exploratory reports on the contralateral side of the operation under the endoscopic view [7, 8], and the incidence of ipsilateral occult hernia under the endoscopic view or during open surgery is rarely reported. No discussion has been made in the literature on the relevant clinical big data of ipsilateral occult inguinal hernia during surgery. Therefore, the incidence and clinical findings of ipsilateral occult hernia during surgery were analyzed and studied with clinical data in this study, to provide references for the diagnosis and treatment of occult inguinal hernia in clinical settings.

## Methods

### Patient selection

This retrospective analysis was conducted on 1066 patients who underwent inguinal hernia surgery at Shanghai Tongren Hospital between January 2016 and October 2018, including 974 men and 92 women, with an average age of 69.32 ± 13.15 years. All patients underwent a detailed preoperative medical history, careful physical examination, ultrasonography or CT examination of the groin, and preoperative informed consent for surgery. The inclusion criteria included: patients whose primary diagnosis was inguinal hernia and underwent a hernia repair operation. The exclusion criteria were: (1) patients with severe cardiopulmonary dysfunction as proven by preoperative examination; (2) patients with oral aspirin or other anticoagulant drugs; (3) patients with abnormal coagulation function before surgery; and (4) patients with an inconsistent intraoperative diagnosis compared with their preoperative diagnosis.

### Descriptions of the expert hernia surgeons (EHS) group and the operation environment

All patients included in this study were operated on under the guidance of senior surgeons (clinical working time > 5 years). The expert hernia surgeons (EHS) group refers to the surgical team specializing in inguinal hernia operation. The open surgical method was Lichtenstein repair or pre-peritoneal space repair (Stoppa operation), while the laparoscopic surgical method was total extraperitoneal herniorrhaphy (TEP). Due to differences in professional level, the EHS group could perform open surgery and endoscopic technique, while the non-expert hernia surgeons (NEHS) group could only perform open surgery.

### Description of occult hernia

A size definition of occult hernia has not been universally adopted worldwide. In the literature, Chinese population studies have introduced the concept of "early" occult hernia as a relatively shallow local pitting of the peritoneum in the inguinal region with a small diameter (approximately < 1 cm) despite the presence of a peritoneum defect. The international guidelines for inguinal hernia from 2018 [1] suggest that hidden hernias should be defined as by the HerniaSurge Group as: asymptomatic hernias not detectable by physical examination.

### Statistical analysis

Values for all continuous variables are expressed as the mean ± standard deviation (SD) of the mean. Data were evaluated by the two-sample t-test for continuous variables and the Chi-square test for categorical variables. Data were analyzed with Origin 8.0 software (Origin Lab Corp., Northampton, MA, USA). *P* < 0.05 was considered a statistically significant result.

## Results

### Patient demographics

Demographic characteristics of occult inguinal hernia cases among the 1066 patients who underwent inguinal surgery were compared and analyzed (Table 1). The incidence of occult inguinal hernia was 8.26% (88/1066) in the surgical population, among which the incidence of occult inguinal hernia was 8.32% (81/974) in the male population and 7.61% (7/92) in the female population. The average age of patients with occult inguinal hernia was 71.68 ± 11.31 years. Among the occult inguinal hernia cases, occult direct inguinal hernia had the highest incidence rate (47/91), followed by indirect inguinal hernia (28/91), femoral hernia (14/91), obturator hernia (1/91), and spigelian hernia (1/91). Occult inguinal hernia was found in 7.48% (27/361) of laparoscopic procedures and in 8.65% (61/705) of open procedures. The incidence of occult inguinal hernia was 8.89% (67/754) in the EHS group and 6.73% (21/312) in the NEHS group. The incidence rate of inguinal hernia on the left side was 7.71% (32/415) and on the right side was 9.89% (56/566). No patients with bilateral occult inguinal hernia were found in the surgical population.

**Table 1:** Demographic characteristics of inguinal hernia cases

Variable	Occult hernia	Non-occult hernia	Total
Patients, n	88*	978	1066
Sex, n			
Male	81	893	974
Female	7	85	92
Ages, years	71.68 ± 11.31 (46–95)	66.22 ± 15.73 (13–98)	
Types of hernia, n	91	1053	1144**
Indirect hernia	28	829	857
Direct hernia	47	183	230
Femoral hernia	14	40	54
Obturator hernia	1	1	2
Spiglian hernia	1	0	1
Operation methods, n	88	978	1066
Endoscopic technique	27	334	361
Open surgery	61	644	705
Operators qualification, n	88	978	1066
EHS group	67	687	754
NEHS group	21	291	312
Location of inguinal hernia, n	88*	978	1066
Left groin	32	383	415
Right groin	56	510	566
Bilateral groin	0	85	85

Values are expressed as the mean ± standard deviation of the mean

*EHS* expert hernia surgeons, *NEHS* Non-expert hernia surgeons

*Three cases among 88 patients who suffered two kinds of occult hernias on the same side. **Different hernias on both sides of the same patient were counted, so the total number of types of hernia (n = 1144) exceeds the total number of patients (n = 1066)

### Comparison of occult hernias between the EHS and NEHS groups

We compared the influence of expert hernia surgeons (EHS) and non-expert hernia surgeons (NEHS) factors on the detection of occult inguinal hernia. Combined, 754 cases of open and endoscopic technique were performed in the EHS group, among which 67 patients were found to have occult hernia (40 cases in open surgery and 27 in endoscopic technique) (Table 2). In the NEHS group, 312 open surgeries were performed, and 21 occult hernias were found. When each subgroup was compared, there was no statistically significant difference in the incidence of occult inguinal hernia between the EHS open surgery group and the NEHS open surgery group (*χ*^2^ = 2.62, *P* > 0.05). There was no statistically significant difference in the incidence of occult inguinal hernia between the EHS endoscopic technique group and the NEHS open surgery group (*χ*^2^ = 0.14, *P* > 0.05). There was no statistically significant difference in the incidence of latent inguinal hernia between the EHS group (open plus endoscopic technique) and the NEHS group (*χ*^2^ = 1.35, *P* > 0.05).

**Table 2:** Comparison of occult hernias between the EHS and NEHS groups

	Occult hernia	Non-occult hernia	*χ*^2^ value	*P* value
EHS group (open)	40	353	*χ*^2^ = 2.62	*P*>0.05
EHS group (Laparoscopic)	27	334	*χ*^2^ = 0.14	*P*>0.05
EHS group (total)	67	687	*χ*^2^ = 1.35	*P*>0.05
NEHS group (open)	21	291		

*EHS* expert hernia surgeons, *NEHS* Non-expert hernia surgeons

### Comparison of occult hernias between open surgery and endoscopic technique

We compared the effects of different surgical methods on the detection of occult inguinal hernia. There were 644 cases of open surgery and 61 cases of occult hernia were found, among which 40 cases were found in the EHS group and 21 in the NEHS group (Table 3). Endoscopic technique was performed in 334 cases, and occult hernias were found in 27 patients. When each subgroup was compared, there was no statistically significant difference in the detection of occult inguinal hernia between the EHS open surgery group and the EHS endoscopic technique group (*χ*^2^ = 1.69, *P* > 0.05). There was no statistically significant difference in the detection of occult inguinal hernia between the EHS endoscopic technique group and the NEHS open surgery group (*χ*^2^ = 0.14, *P* > 0.05). There was no statistical difference in the detection of occult inguinal hernia between the EHS open surgery group and the NEHS open surgery group (*χ*^2^ = 2.62, *P* > 0.05). A comprehensive comparison of occult inguinal hernia detection between the open surgery group and the endoscopic technique group also showed no statistically significant difference (*χ*^2^ = 0.61, *P* > 0.05).

**Table 3:** Comparison of occult hernias between open surgery and endoscopic technique

	Occult hernia	Non-occult hernia	N	*χ*^2^ value	*P* value
Open surgery	61 (8.65%)	644 (91.35%)	705	*χ*^2^ = 0.61	*P*>0.05
Open surgery (EHS)	40 (10.18%)	353 (89.82%)	393	*χ*^2^ = 1.69	*P*>0.05
Open surgery (NEHS)	21 (6.73%)	291 (93.27%)	312	*χ*^2^ = 0.14	*P*>0.05
Endoscopic technique	27 (7.48%)	334 (92.52%)	361		

*EHS* expert hernia surgeons, *NEHS* Non-expert hernia surgeons

### Classification and incidence of occult hernia

There were many types of occult hernias in the groin (Table 4). The incidence of direct inguinal hernia was highest, accounting for 4.11% of all inguinal hernias, followed by indirect inguinal hernia (2.45%), femoral hernia (1.23%), occult obturator hernia, and spigelian hernia, which had the lowest incidence rates in this study (0.08%). There were significant differences among the incidence rates of the different occult hernias (*χ*^2^ = 112.68, *P* < 0.001). When comparing the incidence rates of occult hernias between two groups, a significant difference was observed between occult indirect inguinal hernias and occult direct inguinal hernias (*χ*^2^ = 83.19, *P* < 0.001). A significant difference also existed between occult direct inguinal hernias and occult femoral hernias in the study population (*χ*^2^ = 59.31, *P* < 0.001), but no significant difference was observed between occult direct inguinal hernias and femoral hernias (*χ*^2^ = 0.78, *P* > 0.05). Occult obturator hernias and spigelian hernias were not compared between the groups due to the small number of cases.

**Table 4:** Classification and incidence rate of occult hernia

Types of hernia	Occult hernia	Non-occult hernia	Number	*χ*^2^ value	*P* value
Total	91 (7.95%)	1053 (92.05%)	1144	*χ*^2^ = 112.68	*P*<0.001
Indirect hernia	28 (2.45%)	829 (72.46%)	857	*χ*^2^ = 83.19	*P<0.001
Direct hernia	47 (4.11%)	183 (15.99%)	230	*χ*^2^ = 0.78	^#^*P*>0.05
Femoral hernia	14 (1.23%)	40 (3.52%)	54	*χ*^2^ = 59.31	^△^*P*<0.001
Obturator hernia	1 (0.08%)	1 (0.08%)	2	/	/
Spiglian hernia	1 (0.08%)	0	1	/	/

*P* = occult hernia *vs* non-occult hernia

*P = indirect hernia *vs* direct hernia

^*#*^*P* = direct hernia *vs* femoral hernia

^*△*^*P* = femoral hernia *vs* indirect hernia

### Age and sex factors with occult hernia

According to the analysis of incidences of occult inguinal hernia by gender, the rate of occult inguinal hernia was 8.32% (81/974) in the male surgical population and 7.61% (7/92) in the female surgical population. There was no significant difference in the incidence of occult hernia by gender (*χ*^2^ = 0.06, *P* > 0.05) (Table 5).

**Table 5:** The relationship between sex and occult hernia

Sex	Occult hernia	Non-occult hernia	*χ*^2^ value	*P* value
Male	81	893	*χ*^2^ = 0.06	*P*>0.05
Female	7	85		

Further analysis of occult hernia incidences in different age groups showed that the rates of occult hernias in the inguinal region were significantly different between patients ≤ 50 years and > 50 years (*χ*^2^ = 8.83, *P* < 0.005), with a higher incidence rate in the older group. Meanwhile, with increasing age, the significance of the difference gradually decreased. There was no significant difference in the incidence of occult hernia when the groups were divided by age 70 years (*χ*^2^ = 2.8, *P* > 0.05) (Table 6).

**Table 6:** The relationship between age and occult hernia

Age, years	Occult hernia	Non-occult hernia	*χ*^2^ value	*P* value
≤ 50	2 (2.27%)	130 (13.29%)	*χ*^2^ = 8.33	^a^*P*<0.005
> 50	86 (97.73%)	848 (86.71%)		
≤ 60	14 (15.91%)	276 (28.22%)	*χ*^2^ = 6.18	^b^*P*<0.05
> 60	74 (84.09%)	702 (71.78%)		
≤ 70	43 (48.86%)	568 (58.08%)	*χ*^2^ = 2.8	^c^*P*>0.05
> 70	45 (51.14%)	410 (41.92%)		

^a^*P* =  ≤ 50 *vs* > 50

^b^*P* =  ≤ 60 *vs* > 60

^c^*P* =  ≤ 70 *vs* > 70

### The incidence of ipsilateral occult inguinal hernia in the surgical population

According to the specific incidence of occult hernia and ipsilateral hernia (clinically diagnosed and requiring surgical treatment) in the surgical population, it was found that there were many comorbid conditions (Table 7), among which occult direct hernia with ipsilateral dominant indirect hernia had the highest proportion, accounting for 52.27% of all occult hernias and 43.15% of the surgical population in this study. Occult indirect hernia with ipsilateral dominant direct hernia was second, accounting for 29.55% of occult hernias and 24.39% of the study population. The proportions of other occult hernias combined with the dominant hernia were lower.

**Table 7:** Subgroup classification and incidence rate of ipsilateral occult hernia

Types of occult hernia	Number (n)	Subgroup/occult (%)	Subgroup/overall (%)
		n/88	n/1066^#^
Direct hernia* + Indirect hernia	46	52.27	43.15
Indirect hernia* + Direct hernia	26	29.55	24.39
Femoral hernia* + Indirect hernia	6	6.82	5.63
Femoral hernia* + Direct hernia	4	4.55	3.75
Femoral hernia* + Spiglian hernia	1	1.14	0.93
Indirect hernia* + Funicular hydrocele	1	1.14	0.93
Spiglian hernia* + Direct hernia	1	1.14	0.93
Femoral* + Obturator* + Indirect hernia	1	1.14	0.93
Direct * + Femoral * + Indirect hernia	1	1.14	0.93
Indirect * + Femoral * + Direct hernia	1	1.14	0.93
Total	88	100.00	

* = occult hernia

^#^ = all patients

### The morbidity difference of bilateral occult inguinal hernia

The incidence of occult inguinal hernias on the right side was significantly higher than that on the left (60.44 *vs* 39.56%, *χ*^2^ = 19.42, *P* < 0.001) (Table 8). Further analysis of subdivided groups showed that there was no significant difference in the incidence of occult direct hernia and occult indirect hernia in the bilateral inguinal region (*χ*^2^= 0.88, *P* > 0.05), but the occurrence of occult femoral hernia was significantly different than that of occult direct or indirect hernia (*χ*^2^= 23.91, *P* < 0.001; *χ*^2^= 7.46, *P* < 0.01, respectively). Occult femoral hernia was more common in the left inguinal region (85.71%). Although there was no laterality-based difference in the incidence of inguinal involvement between occult indirect and occult direct hernias, the incidence of the two occult hernias in the right groin (64.29 and 74.47%) was higher than that in the left groin (35.71 and 25.53%). Other rare hernias (obturator hernia and spigelian hernia) were not analyzed due to small sample size.

**Table 8:** Bilateral differences in occult inguinal hernia

Types of occult hernia	Left groin	Right groin	Number (n)	*χ*^2^ value	*P* value
Indirect hernia	10 (35.71%)	18 (64.29%)	28 (30.77%)	*χ*^2^ = 0.88	*P*>0.05
Direct hernia	12 (25.53%)	35 (74.47%)	47 (51.67%)	*χ*^2^ = 23.91	^#^*P*<0.001
Femoral hernia	12 (85.71%)	2 (14.29%)	14 (15.38%)	*χ*^2^ = 7.46	^△^*P*<0.01
Obturator hernia	1 (100%)	0	1 (1.09%)	/	/
Spiglian hernia	1 (100%)	0	1 (1.09%)	/	/
Total (n)	36	55	91(100%)*	*χ*^2^ = 19.42	*P*<0.001

*The number include three cases who suffered two kinds of occult hernias on the same side. *P* = indirect hernia *vs* direct hernia

^#^*P* = direct hernia *vs* femoral hernia

^△^*P* = femoral hernia *vs* indirect hernia

## Discussion

In the development of inguinal hernia, before the hernial sac has become prominent in potential cracks between the groin muscle and fascia, most patients are asymptomatic, physical signs are not obvious, and only a minority of patients (soccer players, weight lifting athletes, heavy manual laborers, etc.) may show symptoms such as dull pain or lower abdomen ache before the hernial sac is present in the groin area. These cases may have occult hernias, because their symptoms and signs are not obvious, so the diagnosis is difficult [9]. Sometimes it is necessary to make a definite diagnosis through detailed medical history and physical examination to exclude other lesions (varicocele, inguinal lymphadenitis, chronic appendicitis, epididymitis, or prostatitis) or through surgical exploration [10]. The 2018 international guidelines for the diagnosis and treatment of inguinal hernia indicate that occult hernia, as defined by the HerniaSurge Group, is an asymptomatic hernia not detectable by physical examination [1]. An occult hernia may exist alone or in combination with other dominant hernias. The occult hernias reported so far in the literature include actual protrusions of normally intra-abdominal contents, a ‘‘beginning’’ hernia, or even just a patent processus vaginalis without herniation [1].

For early potential inguinal hernia, risk factors for the occurrence and development of occult hernia include smoking, family history of hernia, immobilization of the sheath process, collagen metabolism disease, abdominal aortic aneurysm, ascites, peritoneal dialysis, long-term heavy physical activity, and chronic obstructive pulmonary disease. Because the symptoms and signs of occult hernias are atypical, in addition to careful physical examination, other auxiliary examinations are needed to confirm the suspected presence of occult hernias. Although B-ultrasound, computed tomography (CT), and magnetic resonance imaging (MRI) are recommended, their diagnostic sensitivity and specificity are inferior to hernia sac imaging [11, 12]. However, hernia sac imaging is a traumatic test with a risk of visceral injury [10]. The 2009 Guidelines by the European Hernia Society recommend that an ultrasound examination (by a specialist) should be performed first in cases of unexplained swelling and pain in the inguinal area. Only when ultrasound and subsequent MRI are negative (Grade C), is hernia sac imaging recommended.

It has been reported that the incidence of occult hernias in patients diagnosed with unilateral inguinal hernia before surgery was as high as 11–35% [2, 3], and the incidence of contralateral occult hernia during laparoscopic surgery was 11–22% [3, 13]. When bilateral inguinal region exploration was conducted, the incidence of bilateral occult hernias as determined by TEP herniorrhaphy surgery was 5–58% [14, 15], and the incidence of bilateral hidden hernia as determined by transabdominal preperitoneal surgery was 5–58% [4, 8]. The incidence of occult hernia reported in the literature was based on the incidence of occult hernias in the contralateral side during surgery. In this study, we found that the incidence of occult hernias in the ipsilateral side during surgery was 8.26%, which was based on the statistical results of different observation sites. The chapter on occult hernia in the 2018 international guidelines for the diagnosis and treatment of inguinal hernia is mainly based on some reports of contralateral occult hernia found during surgery. Meanwhile, the situation of ipsilateral occult hernia is not described or discussed. Thus, this study provides new evidence-based medical data for the prevention and treatment of occult hernias from different perspectives.

In this study, the epidemiology of occult hernias was studied by analyzing the age, sex, classification and anatomical locations of occult hernias among the surgical population. The results showed that there was no significant difference in the incidence of occult inguinal hernia between men and women, indicating that gender was not a factor affecting the incidence of occult inguinal hernia. Based on the analysis of age factors, there was a significant difference in the incidence of occult hernia between the ≤ 50 group and the > 50 group (*χ*^2^ = 8.83, *P* < 0.005), with a higher incidence of occult hernia in the older group. However, with increasing age, the significant differences were gradually reduced. When the groups were divided by the age cutoff of 70, there was no significant difference in the incidence of occult hernias (*χ*^2^ = 2.8, *P* > 0.05), which indicated that the onset of occult hernias was mainly in older patients. Additionally, with increasing of age, the incidence of occult hernias increased, and the highest incidence rate was observed in patients older than 70 years (9.89%, 45/455, Table 6), indicating that age was an independent factor affecting the occurrence of occult hernias.

Judging from the incidence of occult hernias in the surgical population, any hernia involvement in the inguinal region may be associated with an occult hernia, but there were differences in the incidence of each type of occult hernia; the highest incidence was occult direct inguinal hernia, which accounted for 4.11% of all inguinal hernias, followed by other inguinal hernia (2.45%), femoral hernia (1.23%), occult obturator hernia, and spigelian hernia [9], which was lowest at 0.08%. Because occult hernias exist without a precise degree of incidence, avoiding complications or hernia recurrence is the first thing clinical surgeons must consider. Thus, patients with a primary diagnosis of indirect inguinal hernia should be examined carefully for occult direct inguinal hernia, and patients with a primary diagnosis of direct inguinal hernia should be examined carefully for occult indirect inguinal hernia. Referring to the results of occult hernias found during surgery and follow-up after surgery in previous literature [16–20], for occult hernias on the same side as surgery, careful exploration of potential weak areas in the inguinal region during surgery and complete mesh coverage of all weak areas are the keys to successful surgery.

In this study, we also investigated the advantages and disadvantages of the expert hernia surgeons and non-expert hernia surgeons and the surgical method for detecting occult inguinal hernia. The results indicated that there was no significant difference in the ability of clinically experienced surgeons to detect occult hernias in the surgical population, whether expert or non-expert hernia surgeons, which allowed the results to exclude bias due to differences in surgical ability. In this study, surgery for inguinal hernia involved two approaches: open surgery and laparoscopic repair. Open surgery included Lichtenstein repair or pre-peritoneal space repair (Stoppa operation). The former is primarily used for tension-free repair anterior to the fascia transversalis of the direct inguinal hernia and indirect inguinal hernia, while the latter is used for tension-free repair of the pre-peritoneal space of direct inguinal hernia and femoral hernia. The laparoscopic repair method was TEP surgery, which is the tension-free repair of the total extraperitoneal herniorrhaphy. Generally speaking, for open surgery, occult hernias that are easy to find are mainly direct inguinal hernia, indirect inguinal hernia, and fascial fat hernia. However, for laparoscopic surgery, more types of occult hernias are easy to find, including smaller occult direct inguinal hernia and indirect inguinal hernia, which can be observed under direct vision, and especially smaller occult femoral hernia, which is easier to find using TEP than open surgery. As for why there was no statistically significant difference in the detection of occult inguinal hernia between the open surgery group and the endoscopic technique group, possible reason is that, although the endoscopic technique has a certain advantage on found some occult hernia (femoral and obturatory hernias) than open technology, but occult hernias are mainly direct inguinal hernia and indirect inguinal hernia, the proportion of other occult hernias is very small, when the operations were performed by experienced and attentive surgeons, it is possible that the ability of the two surgical methods to detect all occult hernias is not significantly different. Transabdominal preperitoneal laparoscopic surgery has visibility advantages compared with TEP when detecting occult inguinal hernia, and it can easily detect the situation in the contralateral inguinal area.

For the occult contralateral inguinal hernias found in endoscopic surgeries, the current guidelines are more inclined to repair them [1]. Previous clinical studies have confirmed that if no interventions are made for occult hernias, some will develop into symptomatic hernias and require surgical treatments within a few years after the initial surgeries [21–23]. While the current guidelines do not specifically discuss occult hernias in the ipsilateral side found during open surgery and endoscopic surgery, experienced doctors can solve these problems by covering all the weak areas with large mesh. Considering that occult hernias tend to occur in older patients, failure to carefully explore during the first operation may lead to occult hernias recurring. If an occult hernia requires surgery again several years later, the operation becomes more difficult and risky due to increased age, dysfunction of the heart, lungs, and other important viscera organs, and local anatomical structural damage due to scar adhesion and contracture [5, 6]. As these factors may cause some patients to become unable to undergo surgery, it is necessary for experienced surgeons to carefully detect the possible presence of occult hernia during the operation, especially in elderly patients. Occult hernia is an early stage of clinical dominant hernia, and early detection and treatment are of great significance to improve prognoses [9].

## Conclusions

Occult inguinal hernia has a certain incidence (8.26%) in patients with inguinal hernia. Both ipsilateral and contralateral inguinal hernia may be found during surgery, and careful exploration during surgery should be performed to ensure whether occult inguinal hernias exist. Operations by experienced surgeons and careful detection in elderly patients are essential. For experienced surgeons, both open surgery and laparoscopic surgery can detect occult hernias in the surgical population.

## Data Availability

The datasets used and analysed during the current study are available from the corresponding author on reasonable request.

## Abbreviations

CT: Computerized tomography
TEP: Total extraperitoneal herniorrhaphy
MRI: Magnetic resonance imaging
